# 1-Methyl-D-tryptophan Reduces Tumor CD133^+^ cells, Wnt/β-catenin and NF-κβp65 while Enhances Lymphocytes NF-κβ2, STAT3, and STAT4 Pathways in Murine Pancreatic Adenocarcinoma

**DOI:** 10.1038/s41598-018-28238-8

**Published:** 2018-06-29

**Authors:** Murad Alahdal, Yun Xing, Tingting Tang, Jin Liang

**Affiliations:** 1 State Key Laboratory of Natural Medicines, Jiangsu Key Laboratory of Drug Ability of Biopharmaceuticals, Jiangsu Key Laboratory of Drug Screening, School of life science and Technology, China Pharmaceutical University, Nanjing, China; 2 Medical Laboratory department, Faculty of Medicine and Health Sciences, Hodeidah University, Al Hudaydah, Yemen

## Abstract

1-Methyl-D-tryptophan (1-MT) is extensively utilized in preclinical trials to deplete indoleamine 2,3-dioxigenase (IDO) activity and kynurenine pathway. Since IDO related signaling pathways aren’t well understood, some clinical reports affirmed IDO inhibiting therapeutic significance. Therefore, we did use direct tumor autologous antigens vaccination and 1-MT without chemotherapy to explore biological mechanisms and immunomodulations of 1-MT that motivate antitumor responses. However, DCs antigen-uptake capability, anti-tumor efficiency, intra-tumor and intracellular cytokines were assessed. Besides, CD133+ cells viability and tumor biomarkers were investigated. Splenocytes responses and their signaling pathways such TLRs 2 to 9, NF-κβ1-2, Wnt/β-catenin and TGF-β were dissected. Results evinced that a regimen of 1-MT and TAAs significantly reduced CSC CD133 + viability inside tumor microenvironment, besides increasing tumor cells necrosis and apoptosis. Expression of TGF-β, IDO, RANTES, and PDL-1 was also significantly reduced. Interestingly, 1-MT enhanced lymphocytes TLR2, TLR7, TLR8, and TLR9 pathways. It motivated lymphocytes’ NF-κβ2, STAT3, and STAT4 pathways, while reduced tumors’ NF-κβp65 and Wnt/β-catenin signaling pathways. We found that periphery and intra-tumor Treg cells were significantly decreased. In conclusion, depletion of indoleamine 2,3-dioxigenase activity evidenced IDO relation with tumor stem cells proliferation pathways. Furthermore, 1-MT supports immunotherapeutic vaccines susceptibility and tumor specific targeting by reducing tumorgensis signaling pathways.

## Introduction

Indoleamine 2,3-Dioxygenase checkpoint inhibitors are extensively concerned in the current clinical trials towards several tumor types^1–3^. There are plenty of IDO checkpoint inhibiters, but 1-Methyl-D-tryptophan (1-MT) recently showed promising efficient. It’s a racemic substance blocks kynurenine pathway, and classified as a main responsible of tryptophan and catabolic kynurenine depletion, which selectively impairs T cells proliferation and survival, because T lymphocytes are sensitive to loss tryptophan amino acid^4,5^. Normally, IDO protects maternal tolerance of pregnants’ fetus from T cell responses to MHC-mismatched heterograft^6,7^. New evidences suggest that, IDO becomes highly activated during cancer development^8^. It supports tumor cells escape eradication by immune system^8–10^. Clinical reports concluded that IDO overexpression could polarize APCs to enhance tumor proliferation. Moreover, transfection of tumor cells by IDO gene showed an immunosuppressive activity^11,12^, which glimpses IDOs’ succoring role toward tumor tolerance. The investigation of IDO expression in tumor cells showed that, IDO overexpression into tumor cells is controlled by *Bin1* (tumor suppressor gene), while this gene becomes disabled during tumor development for unknown reasons yet^9^. Therefore, several projects consider IDO work motifs, which could explain clear working itinerary of IDO related pathways to help identification of critical therapeutic target. Because, a lot of human aggressive cancers such as pancreatic, colorectal, lung, ovarian, prostatic, cervical, gastric, head, etc. demonstrated high expression of this enzyme or tryptophan metabolic derivatives^13,14^ during tumor development stage.

Furthermore, a study of IDO-tryptophan catabolism and immune mechanisms in the tumor microenvironment is cloudy and needs more understanding^2^, subsequent studies represented that IDO has multiple immunological functions. It promotes different signaling pathways to suppress T-cell responses, and enhancing Treg cells^15,16^. Collecting all these details together, we find that IDO knockout or inhibition without chemotherapy applications could represent IDO biological signaling pathways in tumor cells and lymphocytes. Nevertheless, IDO relation with immune cells is also complicated and needs deep understanding. It mainly produced from Mesenchymal stromal cells, endothelial cells, fibroblasts, and myeloid-derived antigen-presenting cells^17,18^. Recent reports conducted that mature and immature DCs rather than plasmacytoid DCs could overexpressed IDO in some metastatic cancers^19,20^. Thus signaling pathways involved IDO during tumor related immunomodulations are versatile and poorly understood. Moon Yong *et al*.^21^ revealed that IDO provokes tryptophan shortage, which leads to mechanistic target of rapamycin inhibition (mTORC1) and general control non-depressible 2 (GCN2) activation, that causes anergy of Th1. Meanwhile, bioactive kynurenine pathway stimulates Aryl hydrocarbon receptor (AHR), resulting in promotion of Treg differentiation^21^. Moreover, It was thought that a mechanism of tryptophan degradation happened by base-catalyzed abstraction^22^, while recently was concluded that this mechanism involves formation of a transient ferryl^23^. Whereas, Li, Fang xuan *et al*.^24^ summarized that IDO1 could induces most of immune cells participations. Particularly APCs and cancer related cells through NF-κβ canonical and non-canonical, Jak/STAT pathways, PKC and TGF-β signaling pathways^25^.

Therefore, we blocked IDO activity by using 1-Methyl-D-tryptophan, and used a biological vaccine (tumor autologous antigens) to explore clear evidences regarding virtual work mechanisms and pathways ascribed to IDO activity, without chemotherapy that could confuse results interpretation, because of its harmful side effects^26^. We considered pancreatic adenocarcinoma model in the mice, because recently it’s one of the most aggressive cancers in the world, which showed high rates of IDO overexpression associated tumor aggressiveness^27,28^.

## Results

### TL efficiently activates DCs maturation and T cell during 24 hours

**Figure 1:**
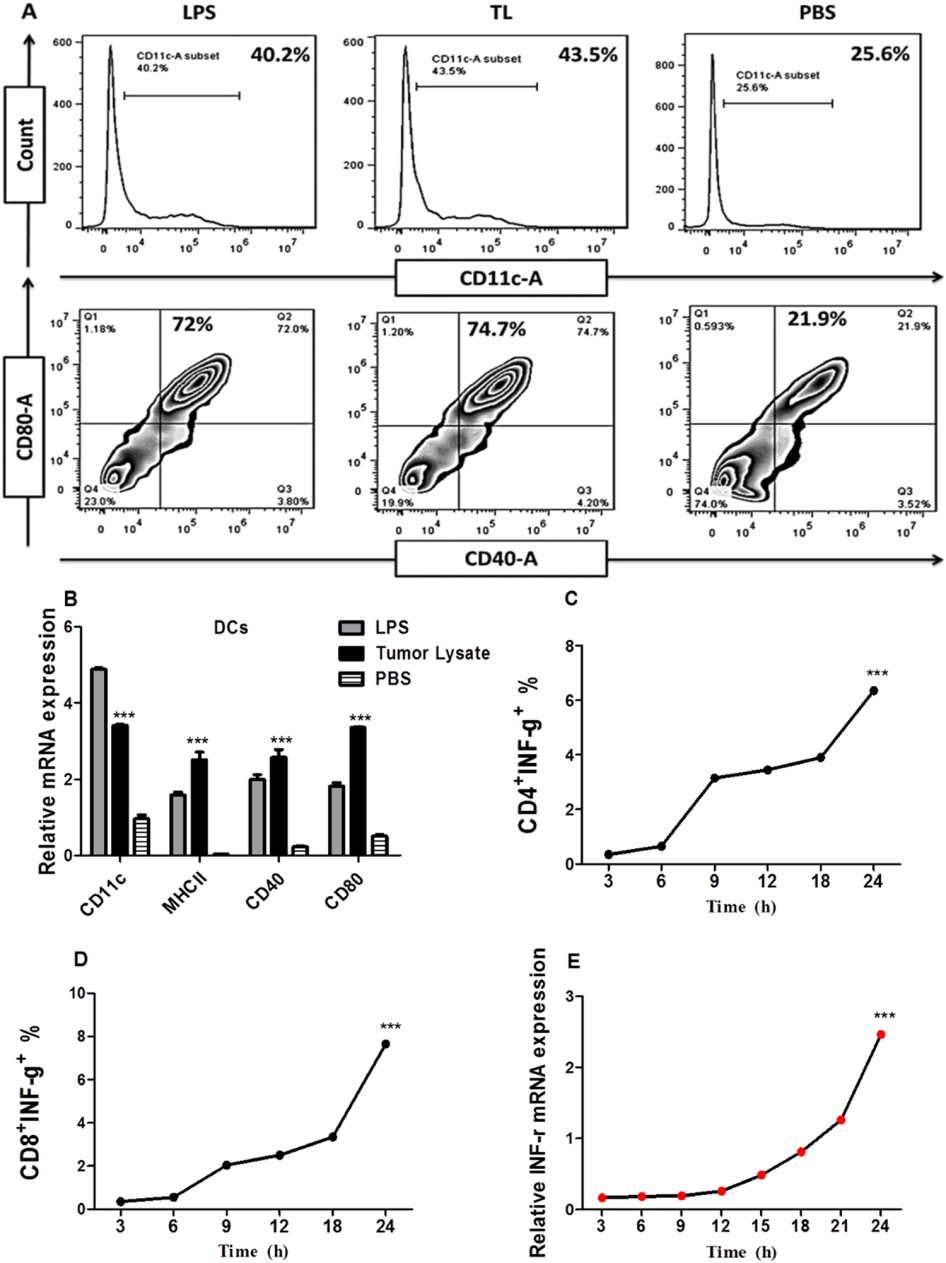
DCs loaded tumor lysate (TL) were tested by Flowcytometer and RT-qPCR to check DCs capability to uptake tumor antigens and present them to Th1 cells. It showed that MHCII, CD40, and CD80 were significantly stimulated by tumor antigens (TL) in comparison to positive and negative controls (**A**,**B**). This is proved that DCs successfully uptake tumor antigens and proceeded presenting potency. Besides that, determination of specific time point to activate Th1 and CTL cells responses by tumor lysate was performed by Flowcytometer interval 3 hours; it showed a highest INF-γ^+^ accumulation at 24 hour, as showed in (**C**), CD4^+^INF-γ^+^ gated from CD3+ significantly raised after 9, 12, and 18 hours, but the highest response showed at 24 hours of incubation. While CD8^+^ INF-γ^+^ activation significantly rose at 18 hours and the highest response was noted at 24 hours of incubation (**D**), these results were confirmed by RT-qPCR to determine a relative INF-γ mRNA expression after activation of splenocytes by (TL = 20 ug/ml). Results showed that high response started at 18 hours to reach a highest at 24 hours (**E**). The statistical significant differences were represented from at least three independent experiments, ***P < 0.001.

The analysis of DCs tumor antigens (TL) uptake potential showed that DCs significantly pulsed in response to tumor lysate in comparison to PBS. It presented high maturation responses; CD40^+^CD80^+^ cells that were gated from CD11c gate represented 74.7% under the impact of TL in comparison to PBS 21.9% (Fig. 1A). Meanwhile, MHCII, CD40 and CD80 genes expression showed significant increasing in comparison to PBS (Fig. 1B); these findings suggested that DCs effectively recognized tumor antigens and efficiently presented to T cell. On the other hand, the determination of optimal time point to activate T cell kinetic and polarization toward tumor antigens showed that accumulation of intracellular INF-γ+ in CD3^+^CD4^+^ cells was increased during 9 hours to reveal 3.15%, but a significant level of INF-γ was detected after 24 hours 6.35% as represented in (Fig. 1C); whereas the accumulation of INF-γ in CD3^+^CD8^+^ cells was also showed significant raising after 24 hours 7.65% as seen in (Fig. 1D). Later, we confirmed these results by analysis *INF-γ*^+^ gene expression in splenocytes by qRT-PCR, which showed a significant expression of *INF-γ*^+^ after 24 hours of incubation (Fig. 1E).

### 1-MT and Tumor lysate inhibit tumorgensis and elicit tumor inflammatory necrosis

**Figure 2:**
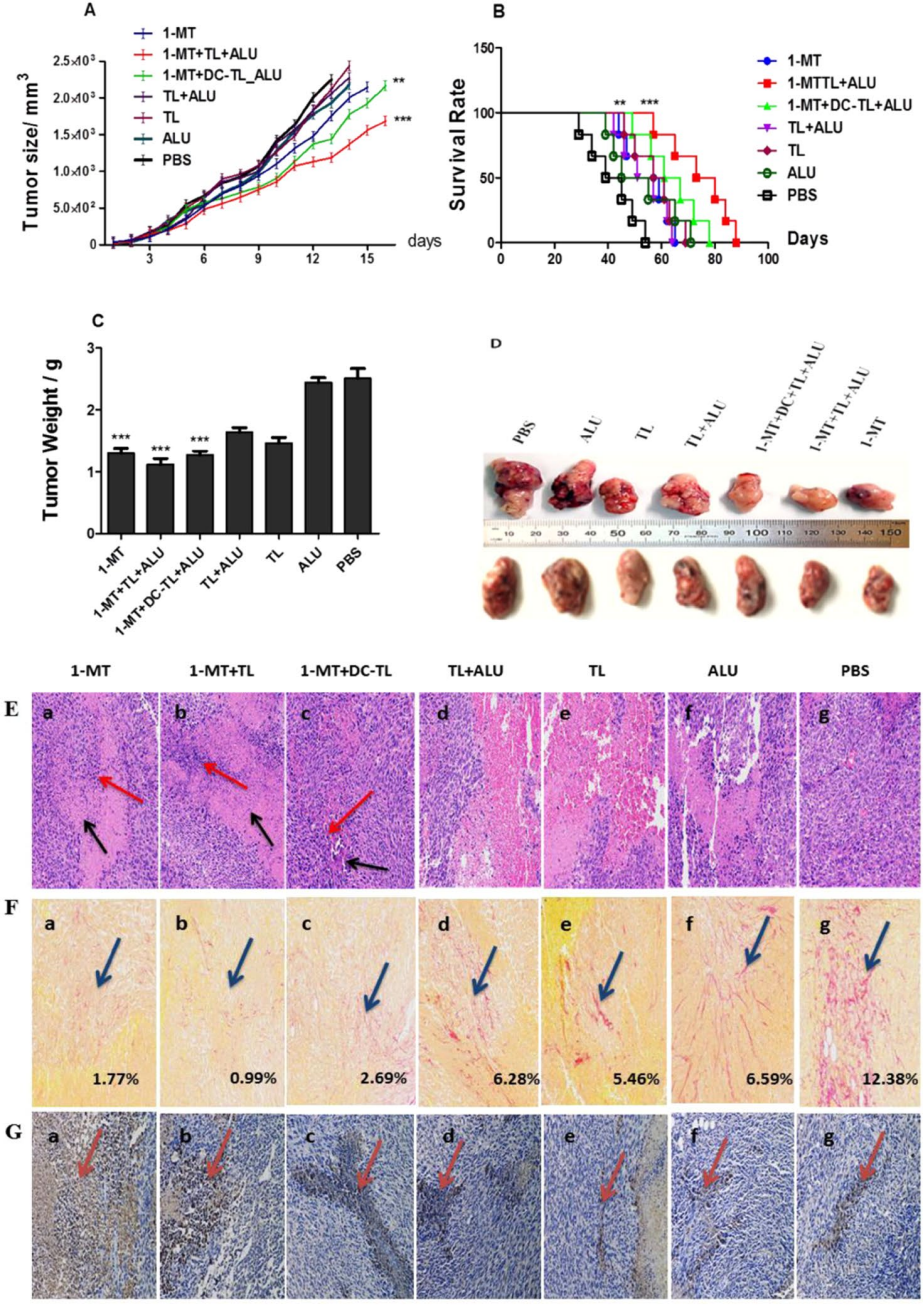
An *in vivo* evaluation of 1-MT+TL/DCs-TL vaccination efficacy in C57BL/6 black mice challenged Pan02 tumor model showed that1-MT and 1-MT+TL/DCs-TL significantly reduced tumor size 2.144 cm^3^, 1.686 cm^3^, 2.166 cm^3^ respectively (**A**), tumor volume and weight also significantly reduced (**C**,**D**). Besides survival rate of 1-MT+TL/1-MT+DCs-TL treated groups was significantly improved (**B**), 38.64%, and 30.77% respectively in comparison with PBS group. Further Haematoxylin and Eosin (H&E) staining showed that, mice treated by 1-MT and 1-MT+TL (**E**.a,b) significantly represented many focal necrosis accompanied with more inflammatory infiltrating cells (black arrow for focal necrosis, and red arrow for inflammatory cells). Meanwhile 1-MT+DC-TL treated group (**E**.c) showed small tumor necrosis and few numbers of inflammatory infiltrating cells. Other tested groups (**E**.d,e,f and g) represented a small focal necrosis inside tumor tissue and a little number of inflammatory cells. The assessment of tumor fibrosis showed that 1-MT, 1-MT+TL, and 1-MT+DCs-TL groups extremely decreased collagen deposition folding rate in treated tumors as shown around pointed area (**F**.a,b,c), while other groups showed high collagen fibers (**F**.d,e,f,g). Besides that 1-MT treated groups showed significant immune reactivity of cleavage caspase3 pathway (**G**.a,b,c) that indicated promoting of tumor apoptosis. While other untreated groups showed no significant apoptotic reactions (**G**.d,e,f,g). These results indicated that administration of 1-Methyl-D-tryptophan and TAA vaccine destroyed tumor stromal and fibrosis, while it promoted significant apoptosis and cell death, which is very interesting observation. ***(*P* < 0.0001), and **(*P* < 0.001).

Subsequently, *in vivo* tumorgensis progression dynamics were significantly inhibited under 1-MT+TL, and 1-MT+DCs-TL regimens effect. We noted that tumor growth of 1-MT, 1-MT+TL, and 1-MT+DCs-TL groups was significantly reduced in comparison to PBS group (2.16 mm^3^/1.30 g, 1.68 mm^3^/1.12 g, 2.14 mm^3^/1.27 g, and 2.5 mm^3^/2.51 g) respectively (Fig. 2A,C,D). Moreover, the survival rate of 1-Mthyl-D-tryptophan treated groups were significantly improved (88days/38.64%, 78days/30.77%) respectively in comparison to PBS (54days) as showed in (Fig. 2B). Furthermore, the analysis of tumor histology showed that 1-MT and 1-MT+TL significantly elicited inflammations and tumor necrosis inside tumor tissue more than 1-MT+DCs-TL (Fig. 2E(a,b,c)). These results indicated that 1-MT effectively enhanced the performance of TL vaccine to elicit lymphocytes responses against tumor. Also, pathologist report described destruction of tumor stromal fibers in groups treated by 1-MT which illustrated an interesting impact of 1-MT on tumor fibrosis.

### 1-MT and 1-MT+TL effectively reduces tumor fibrosis

Tumor fibrosis was critically assessed by using Picro-Sirius red staining technique (PSR) to determine tumor collagen fibers area under 1-MT effect; stained sections showed that 1-MT, 1-MT+TL and 1-MT+DCs-TL groups significantly reduced collagen fibers distribution around tumor tissue in comparison to PBS, it measured 1.77%, 0.99%, 2.69%, and 12.38% respectively (Fig. 2F(a,b,c)). So, collagen fibers deposition were decreased in tumors treated 1-MT. Collagen folding and deposition of PBS group 12.38% ((Fig. 2F(g)) was found higher than 1-MT treated groups that interestingly indicate a high impact of 1-MT on tumor fibrosis.

### 1-MT significantly activates caspase-3 and apoptosis

The impact of 1-MT on tumor cell apoptosis was investigated by testing an immune reactivity of cleaved caspase-3 pathways of 1-MT treated and untreated groups. We found that 1-MT extremely increased caspase3 reactivity in treated tumors (Fig. 2G(a,b,c)), while other groups showed no apoptotic reactions (Fig. 2G(d,e,f,g)). This indicates that 1-MT has a potential role to enhance tumor cell destruction by activating apoptotic signaling pathways.

### 1-MT enhances TILs and significantly reduces CSC

**Figure 3:**
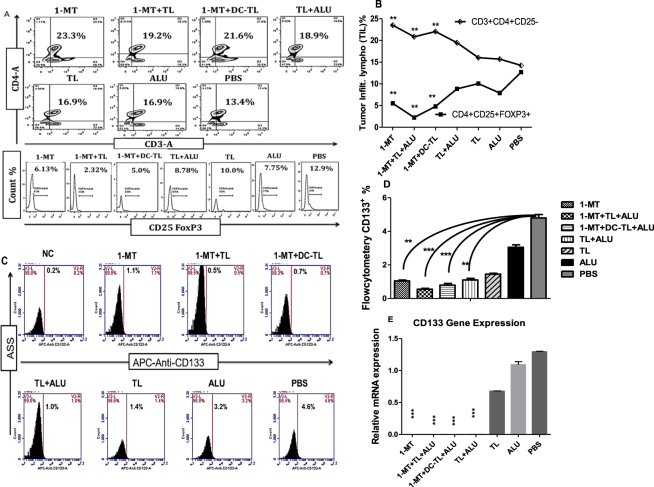
Analysis of tumor infiltrating lymphocytes by Flowcytometer showed that a population of Th1 cells (CD3^+^CD4^+^CD25^−^) was significantly increased inside tumors of 1-MT, 1-MT+TL and 1-MT+DC-TL treated groups in comparison to PBS (**A**,**B**). Meanwhile, Treg^+^ cells (CD25^+^FOXP3^+^) were significantly reduced in comparison to PBS. Cancer stem cells (CSC) CD133^+^ viability in tumor microenvironments of all tested groups showed that 1-MT, 1-MT+TL and 1-MT+DC-TL extremely reduced CD133 viability in comparison to PBS group (**C**,**D**), as well as the analysis of relative mRNA showed that CD133 gene expression was significantly declined in 1-MT+TL and 1-MT+DC-TL treated groups in comparison to PBS group (**E**). These results indicated that 1-MT significantly enhanced TL to elicit immunosurveillance that recognizes and effectively reduces CSC prognosis, which inhibits tumor growth and tumorgensis. ***(*P* < 0.0001), and **(*P* < 0.001).

Further analysis of tumor tissues and microenvironment lymphocytes showed that 1-MT significantly enhanced Th1cells CD3^+^CD4^+^CD25^−^ alone and with TL/DC-TL (23.3%, 19.2%, and 21.6%) respectively in comparison to PBS (13.4%). Meanwhile, it significantly reduced Treg cells CD4^+^CD25^+^Foxp3^+^ in the tumor microenvironment in comparison to PBS group (6.13%, 2.32%, 5%, and 12.9%) respectively (Fig. 3A,B). Whereas a screening of CD133^+^ cells in tumor microenvironment showed that 1-MT, 1-MT+TL, and 1-MT+DCs-TL were significantly reduced a population of CD133^+^ cells in the tumor microenvironment in comparison to PBS (1.0%, 0.5%, 0.7%, and 4.6%) respectively (Fig. 3C,D). These results were ensured by determination of relative CD133 mRNA gene expression among total tumor cells RNA. We found that expression of mRNA was significantly reduced by 1-MT in comparison to PBS (Fig. 3E).

### 1-MT reduces regulatory pathways and enhances tumor inflammatory modulations

**Figure 4:**
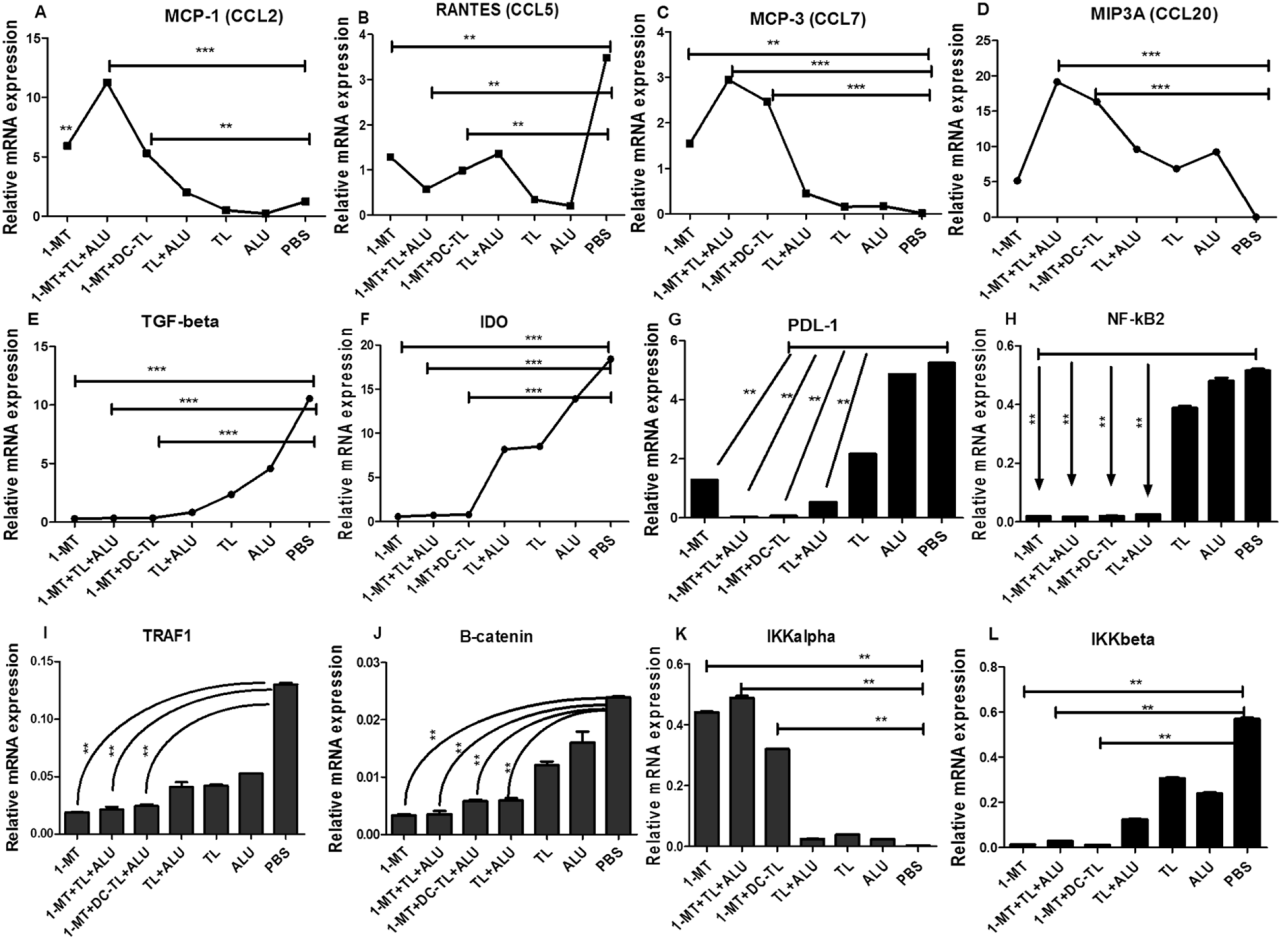
Genetic expression profile of intra-tumor microenvironment cytokines and pathways showed that MCP1, MCP3, and MIP3A were significantly enhanced by 1-MT, 1-MT+TL and 1-MT+DC-TL regimens in comparison to PBS group (**A**,**C** and **D**). Meanwhile, RANTES, TGF-β, IDO, and PDL-1 were significantly reduced in comparison to PBS group (**B**,**E**,**F** and **G**). Further tumor genetic screening showed that NF-κβ2, and β-Catenin pathways were interestingly reduced (**H**,**J**) by1-MT, 1-MT+TL and 1-MT+DC-TL regimens, also TRAF1, and IKKβ mediators were significantly inhibited (**I**,**L**), while IKKα was significantly induced (**K**). These findings clearly evidenced that 1-MT effectively enhanced inflammatory immunomodulations inside tumor microenvironment in response to TL/DCs-TL vaccines, it also reduced tumorgensis enhancers that lead to prevent tumor homing Treg cells as showed in the Fig. 2, which ensured a breakdown of tumor immunotolerance, and recognition of tumor cells by immune surveillance. The statistical significant differences were represented from at least three independent experiments, ***P < 0.001 and **(P < 0.001).

Consequently, relative mRNA assessment of intra-tumor cytokines revealed that 1-MT significantly reduced CCL5 (RANTES) and TGF-β, besides inhibition of IDO enzyme in comparison to PBS as showed in (Fig. 4B,E,F). Nevertheless, 1-MT significantly enhanced inflammatory cytokines CCL2 (MCP-1), CCL7 (MCP-3), and CCL20 (MIP3A) as presented in (Fig. 4A,C and D). The analysis of intra-tumor signaling pathways showed that 1-MT and tumor lysate significantly reduced PDL-1, NF-κβ2, TRAF1, β-Catenin, and IKKβ as presented in (Fig. 4G,H,I,J and L). While, IKKα mediator was significantly activated (Fig. 4K); these results indicated an impact of 1-MT on tumorgensis pathways such as NF-κβ, Wnt/β- catenin, and TGF-β signaling pathways. 1-Mehtyl-D-tryptophan significantly inhibits most of tumor regulatory pathways which do enhance accelerating tumor proliferation and tolerance. These evidences were confirmed by western blot.

### 1-MT and tumor lysate inhibits tumor regulatory pathways

**Figure 5:**
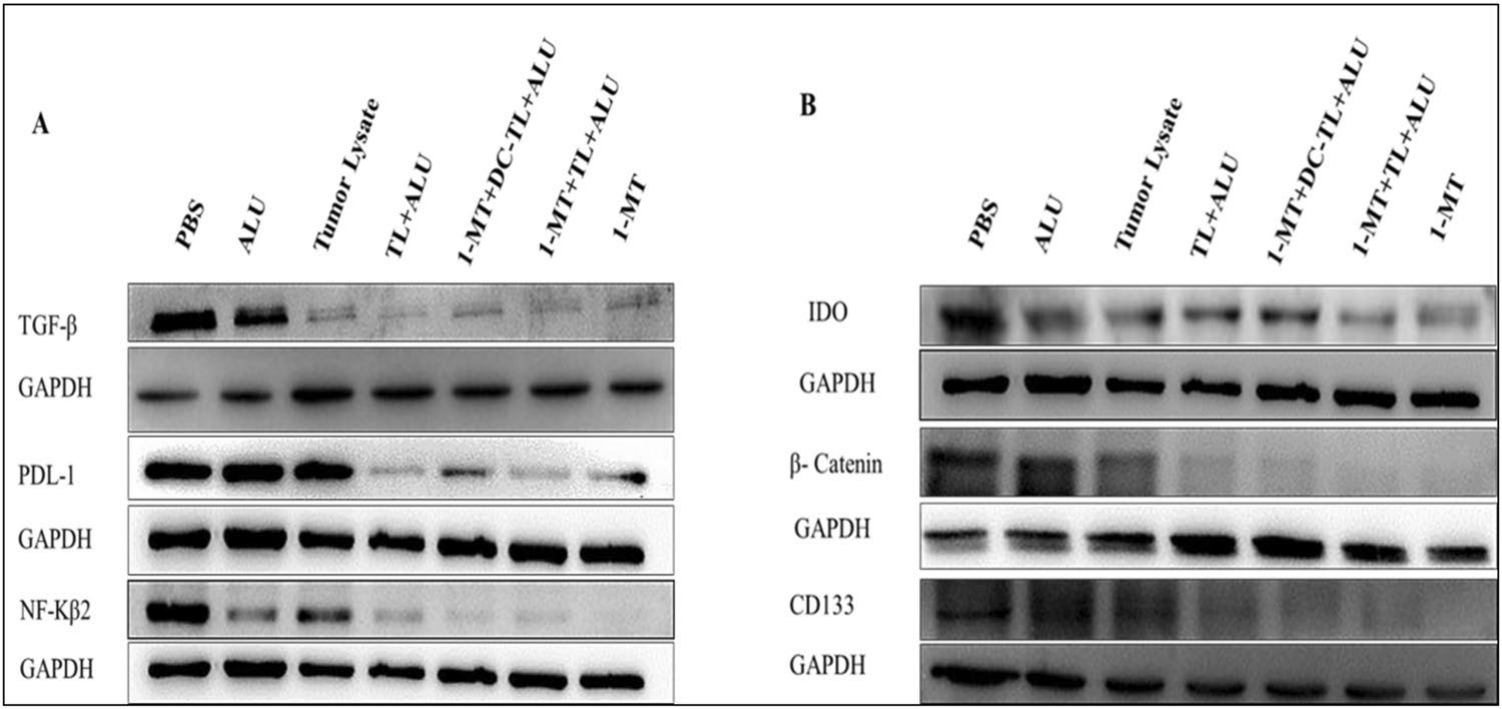
Analysis of tumor microenvironments’ mediators, markers and signaling pathways under the effect of 1-MT-TL/DCs-TL regimens by western blot presented that TGF-β, PDL-1, and NF-κβ2 were significantly inhibited in comparison to PBS (**A**). Meanwhile, IDO, β-catenin, and CD133 showed significant reduction under the effect of 1-MT, 1-MT+TL, and 1-MT+DCs-TL in comparison to PBS (**B**). These results clearly evidenced that 1-MT extremely prohibited main tumor growth, progression, and immune escaping pathways, which activates immune surveillance restore and recruits cellular and humoral immune responses triggering tumor tissues.

Total protein contents of tumor tissues were obtained to evaluate signaling pathways expression; total extracted proteins were loaded by protein loading buffer (PLF) (Blossom, China) for 10 min at 100 °C. Then 5 ug/ml of each protein samples was tested by western blot with Page Ruler Protein marker (Thermo scientific, USA) to test TGF-β, PDL-1, NF-κβ2, IDO, CD133 and β-catenin in comparison to GAPDH positive control. Results showed that 1-MT, tumor lysate, and DCs-pulsed tumor lysate were significantly reduced expression of TGF-β, PDL-1, NF-κβ2, IDO, and β-catenin in comparison to PBS as showed in (Fig. 5A,B). These outputs accumulated more evidences of 1-MT related mechanisms in tumor cells, which confirmed results of genes expression profile screening of intracellular signaling pathways. It interpreted why 1-MT could modulate various humoral and cellular responses.

### Immunofluorescence imaging evidences blocking of NF-κβp65 signaling pathway by 1-MT

**Figure 6:**
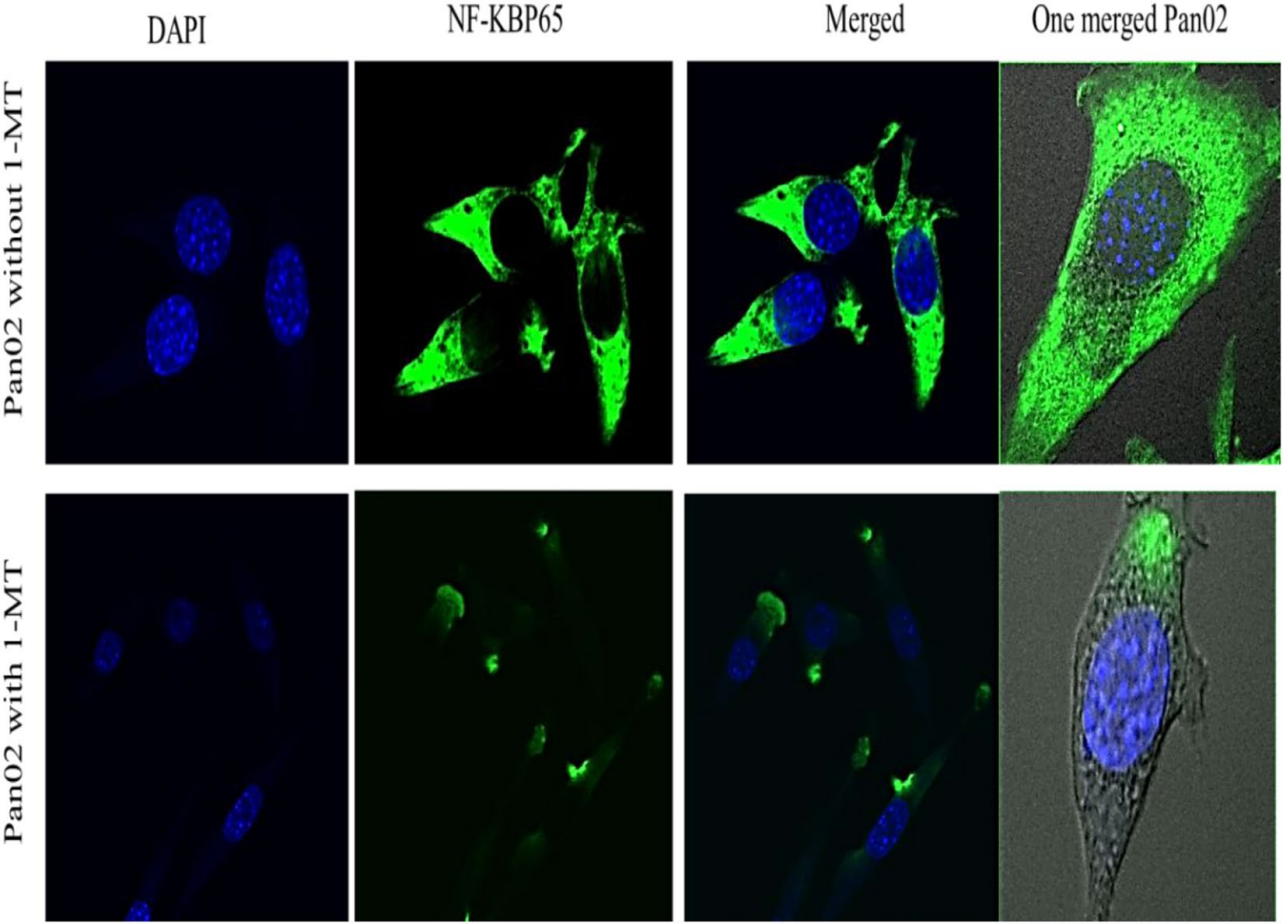
Investigation of NF-κβp65 pathway activity of Pan02 cell line under the effect of 1-MT *in vitro* illustrated that 1-MT effectively inhibited NF-κβp65 in the tumor cells which proved that 1-Methyl-D-tryptophan efficiently depletes tumorgensis activity and forbid tumor immunosuppressive pathways.

Laser imaging of intracellular NF-κβp65 expression of Pan02 tumor cell line which was exposured to 1-MT or non exposured to 1-MT showed that IDO depletion effectively inhibited NF-κβp65 signaling pathway (Fig. 6), that ensured a role of IDO in NF-κβ pathway activation in response to regulatory polarization, the inhibiting of this pathway by 1-MT leads to breakdown tumor tolerance which facilitate modulation of immune responses inside tumor microenvironment.

### 1-MT and TL or DCs-TL promotes splenocytes inflammatory responses

**Figure 7:**
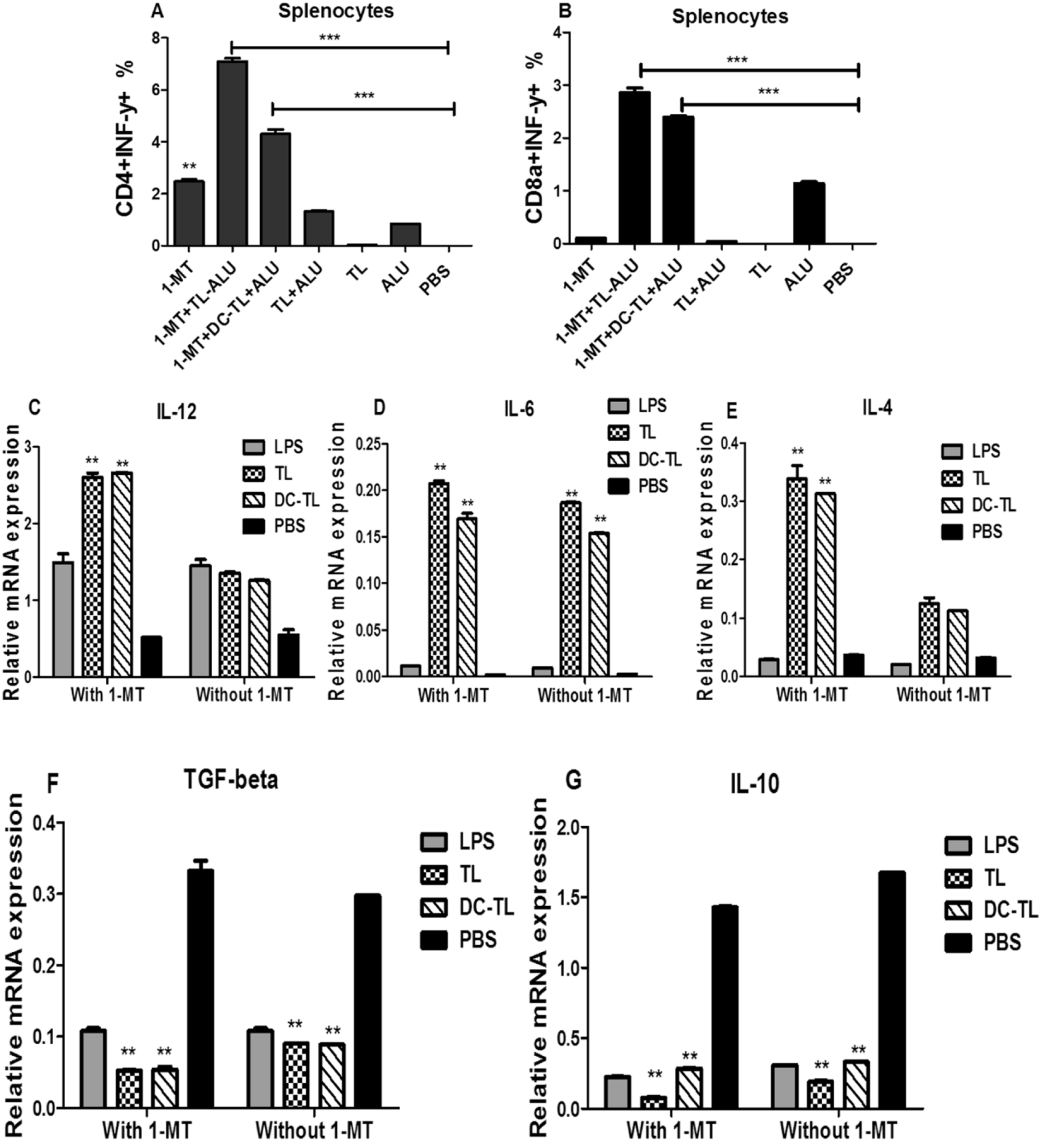
*Ex vivo* lymphocytic kinetic responses of 1-MT treated mice showed that1- significant elicitation of inflammatory responses, it enhanced CD4^+^INF-γ^+^ and CTL INF-γ^+^ accumulation within 24 hours of incubation (**A**,**B**). Besides, the analysis of inflammatory mediators’ relative mRNA expression revealed that 1-MT significantly enhanced IL-12, IL-6, and IL-4 secretion in response to TL/DCs-TL antigens (**C**,**D** and **E**), while TGF-β and IL-10 were significantly reduced in comparison to PBS (**F**,**G**). These results accumulated more evidences confirming specific tumor targeting potential and inflammatory modulations. The statistical significant differences were represented from at least three independent experiments, ***P < 0.001 and **(P < 0.001).

The analysis of CD4^+^ and CD8^+^ T cells’ *ex vivo* kinetics of all immunized groups according to the determined time point (24 hs) showed that 1-MT itself significantly enhanced high accumulation of INF-γ in CD4^+^ cells rather than its efficacy with TL stimulation and also DCs-TL in comparison to PBS group as shown in (Fig. 7A), As well as, CTL (CD8+) cells were also showed significant increasing of INF-γ in response to TL and DCs-TL stimulation in comparison to PBS (Fig. 7B). On the other hand, genes expression of regulatory and inflammatory mediators illustrated that 1-MT elicited increasing of IL-12, 1L-6, and IL-4 expression (Fig. 7C,D,E), while it declined TGF-β, and IL-10 expression (Fig. 7F,G). These results suggested that 1-MT enhanced Th1 CD4+, and CTL CD8+ activation, to initiate proinflammatory immune mediators, while it reduced Treg cells expanding which ascribed to decline of regulatory mediators.

### 1-MT promotes splenocytes’ TLRs signaling pathways in response to TL/DCs-TL

**Figure 8:**
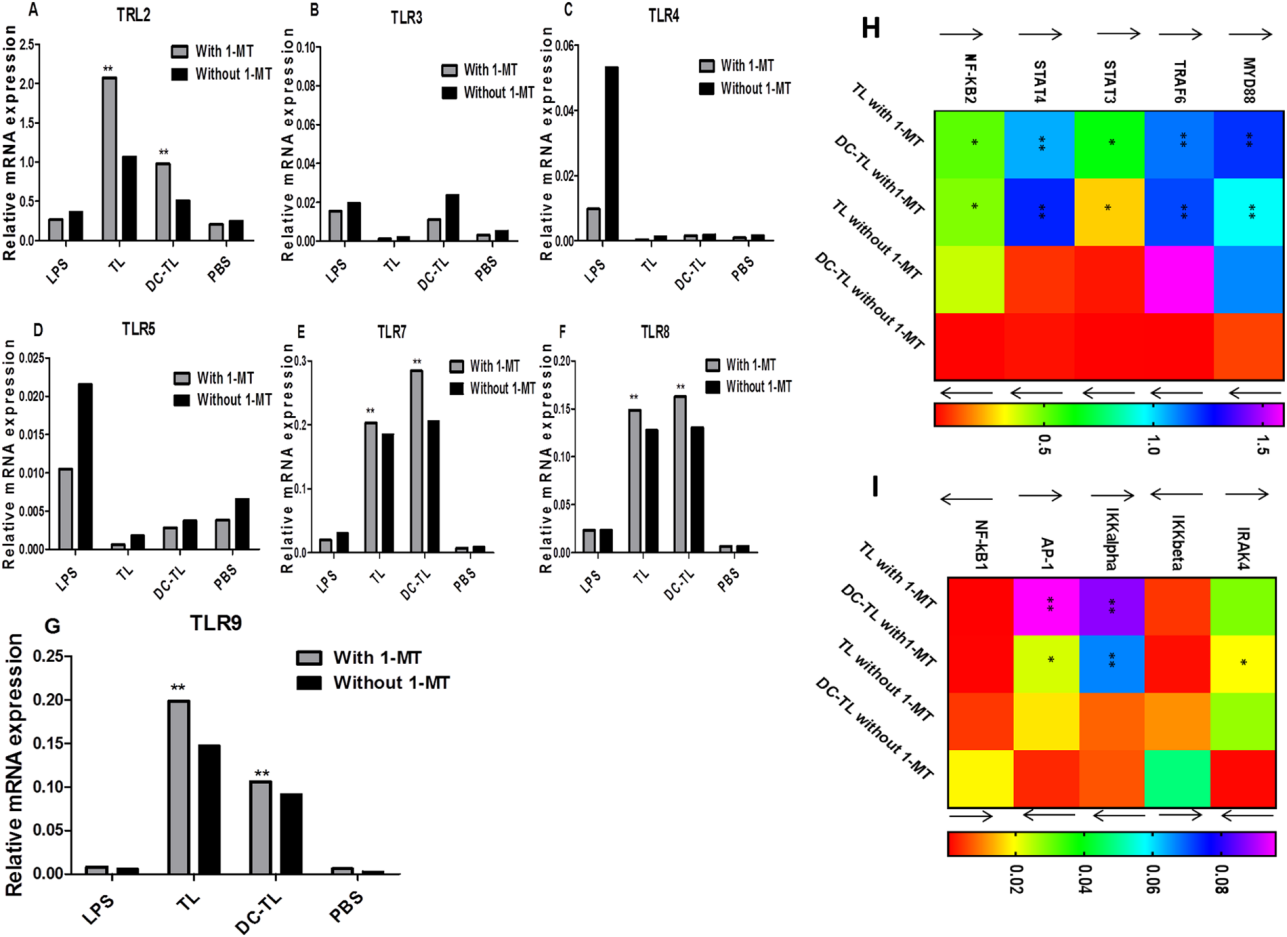
Gene expression profile of splenocytes’ TLRs signaling pathways was determined. Results showed that 1-MT significantly provoked lymphocytic TLR2, TLR7, 8, and 9 in responses to TL/DCs-TL exposure (**A**,**E**,**F** and **9**), while other investigated TLRs showed no responses for TL/DCs-TL effect (**B**,**C** and **D**). Further investigation of intracellular motifs expression profile represented that Myd88, TRAF6, STAT3, STAT4, IRAK4, IKKα, AP-1, and NF-κβ2 mediators were significantly motivated under the effect of 1-MT in response to TL/DCs-TL exposure (**H**,**I**), while regulatory mediators IKKβ and NF-κβ1 were significantly inhibited in comparison to cells without 1-MT. these results showed that 1-MT is the main responsible of immune inflammatory responses to TL/DCs-TL antigens elicitation, that showed no significant responses without 1-MT presence. The statistical significant differences were represented from at least three independent experiments, ***P < 0.001 and **(P < 0.001).

Further splenocytes genes expression profile screening under 1-MT treated groups demonstrated that 1-MT significantly promoted splenocytes’ TLR2, 7, 8, and 9 in comparison to PBS, while TL, DCs-TL also promoted these pathways, but pathways expression was accelerated by 1-MT (Fig. 8A,E,F and G). Meanwhile, other tested TLRs showed no significant responses towards TL, DCs-TL or 1-MT (Fig. 8B,C and D). Furthermore, deep investigation of intracellular pathways showed that 1-MT significantly enhanced an expression of Myd88, TRAF6, IRAK4, STAT3, STAT4, NF-κβ, AP-1, and IKKα, while an expression of IKKβ, and NF-κβ1 was significantly declined (Fig. 8H,I). These results clearly evidenced a role played by IDO towards T cells proinflammatory initiations, which mainly enhances immune regulatory and tumor tolerance through inhibition of immune surveillance functions. Here, 1-MT interestingly facilitated provoking of T lymphocytes immune responses towards tumor antigens.

### Activation of periphery immune surveillance

**Figure 9:**
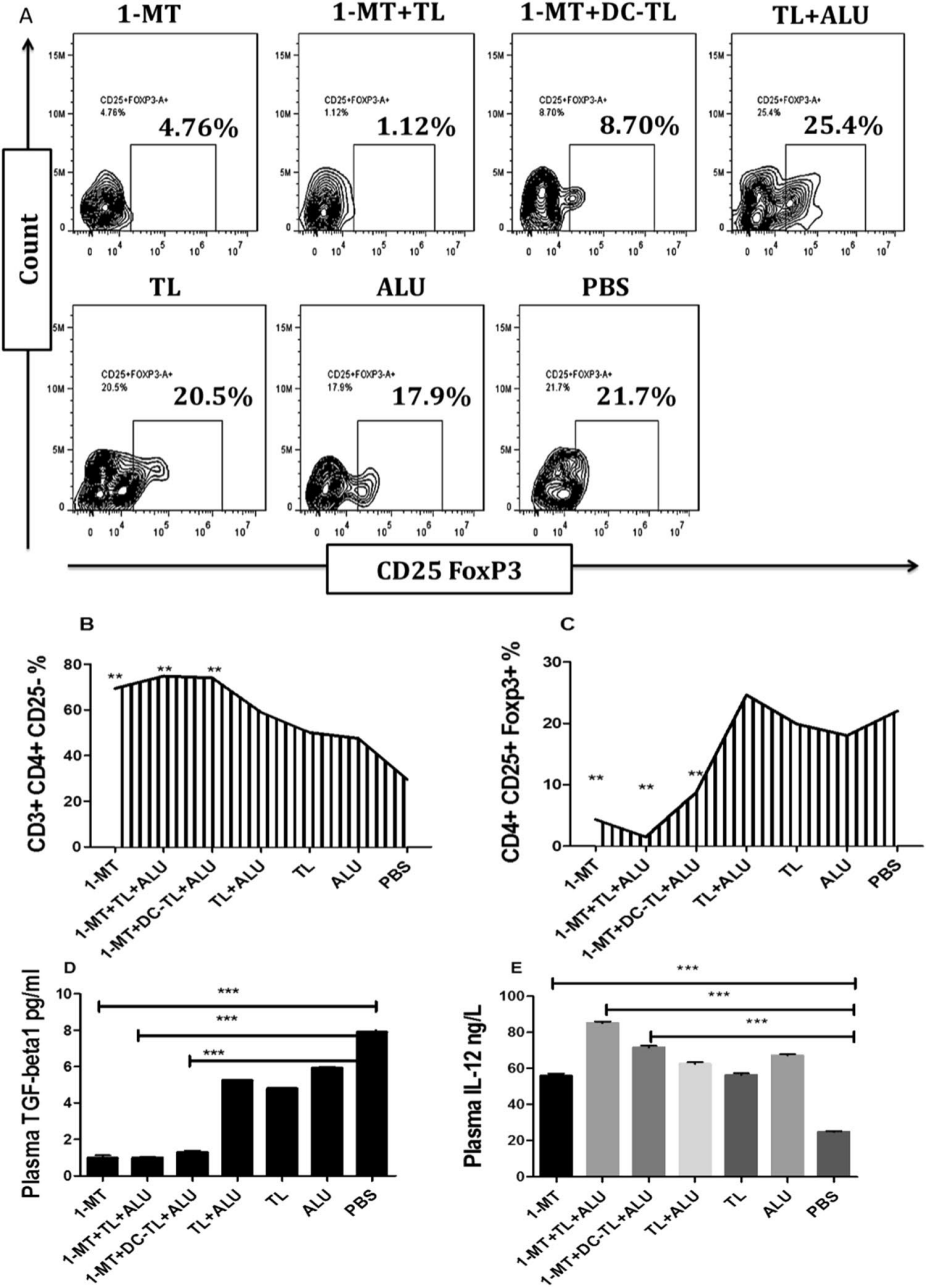
Periphery of cellular and humoral immunological responses of all mice groups illustrated that, administration of 1-MT, 1-MT+TL, and 1-MT+DCs-TL significantly reduced Treg cells population (CD4^+^CD25^+^FOXP3^+^) in comparison to PBS group (4.76%, 1.12%, 8.7%, and 21.7%) respectively (**A** and **C**), while CD4^+^CD25^−^ cells were significantly increased in comparison to PBS (73.9%, 74.7%, 74.2%, and 27.8%) respectively (**A**,**B**). ELISA analysis of periphery cytokines showed that circulating TGF-β was significantly reduced in the mice immunized by 1-MT, 1-MT+TL, and 1-MT+DCs-TL (**D**), while analysis of IL-12 showed significant rising in comparison to PBS (**E**). This is proved that tumor immunosuppressive pathways were completely blocked, while immunosurveillance and inflammatory immunomodulations were efficiently activated. Statistical significant differences were represented from at least three independent experiments, ***P < 0.001 and **(P < 0.001).

Confirmative immunological assessment of periphery immune pathways showed that Treg cells CD25^+^Foxp3^+^ were significantly reduced in the periphery samples of mice treated by 1-MT and TL or DCs-TL in comparison to PBS (4.76%, 1.12%, 8.7%, and 21.7%) respectively (Fig. 9A and C). Meanwhile, CD3^+^CD4^+^CD25^−^ cells were significantly increased in comparison to PBS (73.9%, 74.7%, 74.2%, and 27.8%) respectively (Fig. 9B). Then analysis of periphery cytokines showed that circulating IL-12 was significantly increased at 1-MT, 1-MT+TL, and 1-MT+DCs-TL treated groups in comparison to PBS (55.76, 85.17, 71.53, and 24.8 ng/L) respectively (Fig. 9E). Meanwhile, an investigation of circulating TGF-β showed that periphery TGF-β was significantly declined in mice treated 1-MT, 1-MT+TL, and 1-MT+DCs-TL in comparison to PBS group (0.97, 0.98, 1.26, and 7.90 pg/ml) respectively (Fig. 9D). These findings evidenced that humoral and cellular inflammatory responses were extremely modulated in 1-MT, 1-MT+TL, and 1-MT+DCs-TL treated groups, whereas regulatory mediators were significantly reduced which suggested that tumor tolerance would be broken down and tumor escaping from immunosurveillance would be inhibited.

## Discussion

It’s well-known, that intracellular indoleamine-2,3-dioxygenase IDO by the depletion of tryptophan exerts an immunosuppressive pathways to induce tumor immune escaping^21^. Clinical trials (NCT02077881, NCT00567931) showed that combining IDO inhibitors such as 1-MT with other autologous antigens, chemotherapy, or itself could restore immune immunosurveillance performance^9,29^. To date many cancer regimens were tested, all of them showed limited clinical efficacy, because recruiting T cells against tumor isn’t enough, it becomes functionally inactive when exposure to tumor microenvironment, which was ascribed to overexpression of IDO and regulatory mediators^2,5,30^. Meanwhile, signaling pathways that being orchestrated by IDO mechanisms in tumor microenvironment or myeloid cells are yet poorly understood^21^. Here, we investigated an efficient anti-tumor responses of TL/DCs-TL vaccination, which enhanced by 1-MT performance and its related signaling pathways to understand prognostic IDO work mechanisms related tumor growth, proliferation, and immunosuppressive pathways. We found that IDO mechanisms are complex, there are many signaling pathways mediated by this factor. Blockage of this enzyme enhanced significant humoral and cellular pathways responses to tumor lysate vaccine and/or DCs pulsed tumor lysate, besides significant improvements showed in clinical trials with chemotherapy used 1-MT checkpoint inhibitor^21,31,32^, which clearly evidenced that depletion of IDO activity instantly leads to increase therapeutic susceptibility. Interestingly, we didn’t found documented information about the role of IDO inhibitor on CD133^+^ cancer stem cells (CSC), while our results showed that 1-MT with/without tumor lysate vaccine significantly reduced CD133^+^ cells viability in treated tumor tissues, which suggests that IDO motif role is orchestrated by CSC in the tumor microenvironment. Also, PDL-1 expression was significantly reduced under the effect of 1-MT in comparison to PBS group. This consequently led to inhibit tumorgensis activities, which indicated that cancer stem cells mainly involve IDO pathways to develop tumorigenesis. As the analysis of *in vivo* tumor apoptosis and tumor fibrosis under 1-MT effect showed that 1-MT significantly activates cleaved caspase3 and increased apoptotic potential, while it extremely reduced tumor fibrosis. These findings confirmed a role of IDO in tumor survival and progression, because knocking out IDO activity enhances tumor destruction and promotes tumor apoptosis that illustrates an impact of 1-MT on tumorgensis pathways. This expresses a significance of IDO blockage during tumor therapy. Furthermore, our results accumulated sequential evidences from splenocytes, DLN, and periphery that confirmed a significant promoting role of 1-MT for inflammatory responses and invasion of xenografted tumor models in groups vaccinated tumor lysate, and DCs pulsed TL in case of 1-MT administration, which were on the same line of several studies that investigated likely similar regimens^33–36^. In this study we found that 1-MT significantly reduces tumor cells IKKα, NF-κβ2, and Wnt/β-catenin pathways, besides modulation of its regulatory cytokines in the tumor microenvironment, which significantly inhibited tumor growth and tumor immune escaping potency, that was on the same line of several previous studies^37,38^, but our findings showed that 1-MT significantly enhances IKKα, NF-κβ2, STAT3, and STAT4 pathways of lymphocytes in response to TL/DCs-TL vaccination, which indicated that 1-MT activates intracellular signal transducer, in response to vaccination to induce tumor specific targeting, because activated DCs efficiently trigger presentation of tumor associated antigens to elicit specific tumor targeting. Because we found that TLR2, 7, 8, and 9 were significantly activated in the splenocytes of TL/DCs immunized mice during 1-MT addressing, which elucidated non-enzymatic activity of IDO engage tumorigenesis proceeds. These evidences apparently revealed that 1-MT completely forbids tumor tolerance pathways, which interprets the reduction of regulatory mediators and Treg cells in the tumor microenvironment and periphery analysis. These analyses represented a virtual antitumoral kinetics during vaccination period, which provided some biological evidences that could optimize solid tumor treatment strategies in the near future

## Conclusion

We conclude that, our results revealed that 1-MT has a potential to breakdown tumor tolerance and significantly releases immunosurveillance potential throughout depletion of kynurenine related pathways, besides activation of functional DCs and specific T cells to recognize tumor antigens and activate inflammatory modulations efficiently. These processes involve enzymatic and non-enzymatic mechanisms that need deep analysis of metabolic products pathways as a fundamental secret to identify specific motifs could lead to design high critical therapeutic strategies. However, tumor autologous antigens vaccine with 1-MT showed a significant therapeutic potential as an immunotherapeutic vaccine towards pancreatic adenocarcinoma in the murine models, which encourage this strategy implementation in human clinical trials.

## Materials and Methods

### Mice details and Ethical Issue

Black females (20–25) g C57BL/6 mice were recruited from the Laboratory Animal Center of Yangzhou University. Experimental animals were housed in plastic cages under pathogen-free conditions. Experiments were performed in accordance with the National Institute of Health Guide for the Care and Use of Laboratory Animals, meanwhile it approved by the Institutional Animal Care and Use Committee of China Pharmaceutical University.

### Preparation of Tumor lysate (TL) and DCs loaded TL

Pan02 cell line was utilized to prepare freeze–thawed tumor lysate, as described by W. Asavaroengchai *et al*.^39^. Then protein concentration of supernatant was determined by BCA protein assay kit (Beyotime, China), according to the manufacturer instructions. However, 20 ng/ml of tumor lysate was introduced to (1 × 10^6^) bone marrow dendritic cells (DCs) for 24 hours, in comparison to 1 ng/ml LPS and PBS. Later, pulsed DCs were stained by APC-Anti-mouse anti-CD11c, FITC-Anti-mouse anti-CD40, and PE-Anti-mouse Anti-CD80 (Biolegend, USA) for 30 min at 4 C. Thereafter, DCs uptake capability was assessed by BD Accuri C6 Flowcytometer (Biosciences, USA). On the other hand, to ensure activation status of DC loaded tumor lysate, on the 6^th^ day DCs were co-cultured with TL, as described before for 24 hours and then total RNA was extracted by TRizol (Ambion, USA). Consequently, 500 ng/ul of RNA/sample was mixed with 4 ul of 5x All- In- one RT master mix (Abm, USA), and then final volume was completed to 20 ul. After that, RNA was reversed by reverse transcriptase to cDNA (incubate 25 °C for 10 min, then worm 42 °C for 30 min. Thereafter, reaction was stopped at 85 °C for 5 min and chilled at 4 °C) by thermo cycler (Thermofisher scientific, Finland). Later, cDNA samples were amplified by light cycler 480 II qPCR (Roche, USA) with 2x SYBR Green qPCR master mix (Bimake, China), CD11c (Itgax), MHCII (H2-Ab1), CD80, CD40 primers (Anhui, China), and cyc positive control gene which listed in the Table 1. Then gene expression was calculated according to cyc gene ct.

**Table 1:** Characterization of DCs Primers used for RT-qPCR.

Gene	NCBI Gene ID	Orientation	Primer sequence (5′->3′)	Amplicon Size
Cd11c/Itgax	16411	Forward	CTGGATAGCCTTTCTTCTGCTG	113
		Reverse	GCACACTGTGTCCGAACTCA	
MHCII/H2-Ab1	14961	Forward	AGCCCCATCACTGTGGAGT	66
		Reverse	GATGCCGCTCAACATCTTGC	
CD40	21939	Forward	TGTCATCTGTGAAAAGGTGGTC	120
		Reverse	ACTGGAGCAGCGGTGTTATG	
CD80	12519	Forward	ACCCCCAACATAACTGAGTCT	102
		Reverse	TTCCAACCAAGAGAAGCGAGG	
Cdk1	12534	Forward	AGAAGGTACTTACGGTGTGGT	128
		Reverse	GAGAGATTTCCCGAATTGCAGT	

### Determination of T cell activation time point under TL effect

C57BL/6 black mice were immunized by TL, and then splenocytes were harvested to separate T lymphocytes according ot density. Then separated lymphocytes were co-culture with TL for 3, 6, 9, 12, 18, and 24 hours in the presence of Brefeldin A (BFA) 1,000x (Biolegend, USA) in comparison to ConA, and PBS. Next lymphocytes were collected, washed and stained (surface and intracellular) as described by Wei Ye *et al*.^40^, by using Pecp-Anti-mouse anti-CD3, FITC-anti-CD4, and PE- anti-CD8 for 30 min on ice. After fixation and permeabilization intracellular staining was performed by APC-anti-INF-γ for 30 min on ice. Finally cells were assessed by BD Accuri C6 Flowcytometer. Further confirmation test of specific time points were performed by RT-qPCR. However, total RNA of lymphocytes-TL exposured was extracted by TRizol, and then RNA transcriptase was performed to get cDNA as described before, then it used to determine relative INF-γ mRNA according to forward and reverse primers (Anhui, China) listed in the Table 2.

**Table 2:** Characterization of Tumor Markers Primers Used for RT-qPCR.

Gene	NCBI Gene ID	Orientation	Primer sequence (5′->3′)	Amplicon Size
Ifng	15978	Forward	ATGAACGCTACACACTGCATC	182
		Reverse	CCATCCTTTTGCCAGTTCCTC	
Prom1	19126	Forward	CTCCCATCAGTGGATAGAGAACT	81
		Reverse	ATACCCCCTTTTGACGAGGCT	
Cd274	60533	Forward	GCTCCAAAGGACTTGTACGTG	238
		Reverse	TGATCTGAAGGGCAGCATTTC	
Gapdh	14433	Forward	AGGTCGGTGTGAACGGATTTG	123
		Reverse	TGTAGACCATGTAGTTGAGGTCA	

### Immunization and *In Vivo* Tumor Evaluation

Black C57BL/6 mice were divided 7 groups (n = 6). They were subcutaneously challenged on the right flank by 1 × 10^5^ Pan02 cell line. 6 days later, 1-MT was orally administrated (2 mg/ml)/mouse interval 2days, as described by several protocols^24,31,41^ to the first three group’s 1-MT, 1-MT+TL+ALU, and 1-MT+DC-TL+ALU. Other groups TL+ALU, TL, ALU, and PBS were treated without 1-MT. However, mice were immunized three times subcutaneously with tumor lysate (100 ng/ml) that was mixed with adjuvant (Al(OH)3 aluminum hydroxide gel). This mixture was connected with Polyethylenimine (PEI 5 mg/ml) particles. Then it was kept at 4 °C for 30 min, thereafter, it was subcutaneously injected into mice. During the immunization programme, tumor size of all mice groups was daily measured. Thereabout, mice survival rate of another 7 groups was daily noted until the death of last mouse. However, tested groups were sacrificed and their tumor tissues, blood, spleens and draining lymph nodes were collected.

### Tumor Infiltrating Lymphocytes (TIL) and HE staining

To investigate tumor necrosis and tumor infiltrating T lymphocytes CD4^+^CD25^−^ in comparison to CD4^+^FOXP3+, tumor samples were sectioned 4 µm and introduced to Haematoxylin and Eosin (H&E) staining protocol according to Fischer *et al*.^42^. Thereafter, results were numbered and sent to a pathologist for consultation without any details about the treated and untreated tissues. Finally, tumor tissues were visualized by light microscope 200x. Furthermore, samples of each fresh enucleated tumor were mechanically digested to get single cell suspension. Next cells were counted and recruited to surface and intracellular staining. Briefly, 1 × 10^6^ cells of tumor tissue/sample was firstly stained by Percp-anti-mouse Anti-CD3, FITC-Anti-CD4, and PE-Anti-CD25 for 30 min on ice. Then cells were fixed, permeabilized and stained by APC-Anti-FoxP3 for 30 min on ice. Finally cells were assessed by BD Accuri C6 Flowcytometer.

### Picro Sirius Red Staining

To evaluate the impact of 1-MT and 1-MT+TL on tumor fibrosis, sections of enucleated tumor tissues were deparaffinized, rehydrated and processed for Sirius red staining as described by Segnani *et al*.^43^. Briefly, fixed slides were co-incubated with 0.1% Sirius red aqueous dye solution (Abcam, USA) for 1 hour. Then slides were washed in acidified water. Later it was dehydrated and mounted with DPX. Collagen red fibers and fibers area were measured by Image-pro plus 6.0 (Media Cybernetics, Inc., Rockville, MD, USA).

### Immunohistochemistry analysis of cleaved caspase-3 pathway

To assess an impact of 1-MT and 1-MT+TL on tumor apoptosis potency, fixed tumors were deparafinzed and rehydrated by ethanol, endogenous peroxidase activity was blocked by soaking in absolute methanol including 0.3% H_2_O_2_ for 30 min. then antigen was retrieved by using 10 mmol/L Tris-HCl buffer (pH 8.0) containing 1 mmol/L EDTA. Later it was blocked with normal goat serum for 5 min. Thereafter, anti-activated caspase3 antibody which was diluted 1:20 (Cell signaling, USA) was performed for overnight. Next tissues were washed by TBS and incubated with goat anti-rabbit IgG conjugated with rhodamine for 30 min. Finally they were visualized by light microscope at 200x magnification.

### Determination of cancer stem cells (CSC) viability in treated and non-treated tumor tissues

Single cell suspensions were utilized to determine viability of CSC population in 1-MT treated and non-treated groups. However, 1 × 10^6^ tumor cells were washed and stained by APC-Anti-mouse Anti-CD133 for 30 min on ice, and then stained cells of all groups were tested by BD Accuri C6. To confirm these results, tumor CD133 gene expression was assessed by RT-qPCR according to purchased primers (Anhui, China) listed in the Table 2 as described before.

### Genetic expression profile of intra tumor microenvironment cytokines and pathways

Collected tumor tissues were digested to obtain total RNA for screening CCL2 (MCP-1), CCL5 (RANTES), CCL7 (MCP-3), CCL20 (MIP3A), TGF-β, IDO, PDL-1, TRAF1, IKKα, IKKβ and β-Catenin genes expression by the same method described above. All used primers (Anhui, China) were listed in the Table 3.

**Table 3:** Primers of Splenocytes and Tumor Cytokines used for RT-qPCR.

Gene	NCBI Gene ID	Orientation	Primer sequence (5′->3′)	Amplicon Size
Ccl2	20296	Forward	TTAAAAACCTGGATCGGAACCAA	121
		Reverse	GCATTAGCTTCAGATTTACGGGT	
Ccl5	20304	Forward	GCTGCTTTGCCTACCTCTCC	104
		Reverse	TCGAGTGACAAACACGACTGC	
Ccl7	20306	Forward	GCTGCTTTCAGCATCCAAGTG	135
		Reverse	CCAGGGACACCGACTACTG	
Ccl20	20297	Forward	GCCTCTCGTACATACAGACGC	146
		Reverse	CCAGTTCTGCTTTGGATCAGC	
Tgfbr1	21812	Forward	TCTGCATTGCACTTATGCTGA	100
		Reverse	AAAGGGCGATCTAGTGATGGA	
Ido1	15930	Forward	GCTTTGCTCTACCACATCCAC	170
		Reverse	CAGGCGCTGTAACCTGTGT	
Il12b	16160	Forward	TGGTTTGCCATCGTTTTGCTG	123
		Reverse	ACAGGTGAGGTTCACTGTTTCT	
Il10	16153	Forward	GCTCTTACTGACTGGCATGAG	105
		Reverse	CGCAGCTCTAGGAGCATGTG	
Il6	16193	Forward	TAGTCCTTCCTACCCCAATTTCC	76
		Reverse	TTGGTCCTTAGCCACTCCTTC	
Il4	16189	Forward	GGTCTCAACCCCCAGCTAGT	102
		Reverse	GCCGATGATCTCTCTCAAGTGAT	

### Analysis of tumor microenvironments’ signaling pathways by western blot

First, red blood cells were disrupted, and then tumor cells were washed and fractured by using RIPA buffer (Thermo fisher, USA) with phenyl methyl sulfonyl fluoride (PMSF) a proteinase inhibitor (Sigma, USA) according to the manufacturer instructions. Total protein was assessed by BCA to determine protein concentration for every sample. Thereafter, 5 ug/ml of each protein sample was utilized to test TGF-β, IDO, PDL-1, CD133, NF-κβp65, and β- Catenin pathways expression by western blot. Briefly, samples were run on 10% gel, and then transfer on blotting membrane for 30 min by Trans-blot Turbo (Bio-Rad, Singapore). After milk blocking, membranes were incubated with primary antibodies Anti-TGF-β (Abcam, USA), Anti-IDO (Biolegend, USA), Anti-PDL-1, Anti-NF-κβp65 (Abcam, USA), Anti-CD133 (Boster, China), and Anti-β- Catenin (Affinity, USA) for overnight on shaker, and then washed three times and incubated with goat anti-rabbit secondary antibody (Abcam, USA) for 1–2 hours. Finally, results visualized by gel imaging system 5200 (Tanon, China).

### Immunofluorescence imaging of tumor NF-κβp65 pathway with/without 1-MT

As described by Katsori *et al*.^44^, NF-κβp65 signaling pathway was determined in murine pancreatic cancer cell line (Pan02) under the effect of 1-MT or without 1-MT. briefly, Pan02 cells were cultured in RPMI 1640 (Key GEN Bio, China) supplemented by 10% Fetal Bovine Serum (Hycone, Australia), and 1% penicillin and streptomycin (Gibco, USA) for 24 hours. Then 1 × 10^4^ cells co-cultured with/without 10 ng/ml of 1-MT. 24 hours later cells were washed with phosphate-buffered saline (PBS) then cells fixed and permeabilized by 4% formaldehyde in PBS. Next cells were blocked in 5% milk–10%, Triton X-100 in PBS for 1 h at room temperature. Then p65 visualized by purified Anti-NF-κβp65 (ab32536) at 1:100 dilutions for 12 hours. After that cells were washed and probed by 1:500 dilution of Alexa Fluor 488 goat anti rabbit IgG for 2 hours. Later, DAPI nuclear counterstain was performed for 10 min. Finally, cells were washed by PBS and scanned by laser confocal microscopy FV1000-ASW (Olympus, Japan).

### Intracellular kinetics of splenic CD4^+^ and CD8^+^ T Cells

Splenocytes and draining lymph nodes DLN of the 7 immunized groups were used to investigate intracellular kinetics by determination of T CD4^+^ INF-γ^+^, and CTL^+^ INF-γ^+^ cells. However, lymphocytes were stained for surface markers and intracellular for INF-γ as described above. Harvested results were presented by graph pad software. Thereafter, all CD3^+^CD4^+^ and CD3^+^CD8^+^ cells were collected in separate tubes by FACS Area Flowcytometer. These collected cells were fractured by TRizol to collect total RNA, which used to prepare cDNA by which expression of lymphocytes intracellular cytokines was determined under the effect of 1-MT. However, genes expression of IL-12, IL-6, IL-4, TGF-β, and IL-10 was quantitatively determined as described above with primers (Anhui, China) listed in the Table 3.

### Gene expression profile of splenocytes’ TLRs signaling pathways ascribed to 1-MT

To determine splenocytes activated signaling pathways under 1-MT effect, spleen and NLD cells were co-cultured with tumor lysate, DCs-pulsed tumor lysate, LPS, and PBS with or without 1-MT for 24 hours at 37 °C. Then total RNA was obtained, thereafter TLR2, 3, 4, 5, 7, 8, and 9 relative mRNAs were assessed by RT-qPCR by the same method was mentioned above, upon the primers (Anhui, China) listed in the Table 4.

**Table 4:** Primers of TLRs Pathways used for RT-qPCR.

Gene	NCBI Gene ID	Orientation	Primer sequence (5′->3′)	Amplicon Size
Tlr2	24088	Forward	GCAAACGCTGTTCTGCTCAG	231
		Reverse	AGGCGTCTCCCTCTATTGTATT	
Tlr3	142980	Forward	GTGAGATACAACGTAGCTGACTG	162
		Reverse	TCCTGCATCCAAGATAGCAAGT	
Tlr4	21898	Forward	ATGGCATGGCTTACACCACC	192
		Reverse	GAGGCCAATTTTGTCTCCACA	
Tlr5	53791	Forward	GCAGGATCATGGCATGTCAAC	130
		Reverse	ATCTGGGTGAGGTTACAGCCT	
Tlr7	170743	Forward	CACCACCAATCTTACCCTTACC	76
		Reverse	CAGATGGTTCAGCCTACGGAA	
Tlr8	170744	Forward	GAAAACATGCCCCCTCAGTCA	109
		Reverse	CGTCACAAGGATAGCTTCTGGAA	
Tlr9	81897	Forward	ATGGTTCTCCGTCGAAGGACT	118
		Reverse	GAGGCTTCAGCTCACAGGG	

Later, tracking of splenocytes intracellular signaling motifs ascribed 1-MT activity was performed. However, genes expressions of Myd88, TRAF6, IRAK4, Ikkβ, Ikkα, AP-1, NF-κβ1, NF-κβ2, STAT3, and STAT4 were evaluated as detailed before upon the primers (Anhui, China) listed the Table 5.

**Table 5:** Primers of Signaling Pathways used for RT-qPCR.

Gene	NCBI Gene ID	Orientation	Primer sequence (5′->3′)	Amplicon Size
Myd88	17874	Forward	TCATGTTCTCCATACCCTTGGT	175
		Reverse	AAACTGCGAGTGGGGTCAG	
Traf1	22029	Forward	AGGGTGGTGGAATTACAGCAA	194
		Reverse	GCAGTGTAGAAAGCTGGAGAG	
Traf6	22034	Forward	AAAGCGAGAGATTCTTTCCCTG	125
		Reverse	ACTGGGGACAATTCACTAGAGC	
Irak4	266632	Forward	CATACGCAACCTTAATGTGGGG	125
		Reverse	GGAACTGATTGTATCTGTCGTCG	
Jun	16476	Forward	TTCCTCCAGTCCGAGAGCG	133
		Reverse	TGAGAAGGTCCGAGTTCTTGG	
Ikbkb	16150	Forward	ACAGCCAGGAGATGGTACG	297
		Reverse	CAGGGTGACTGAGTCGAGAC	
Ikbkap	230233	Forward	CTGAAGTTGCATCGGACCCTG	78
		Reverse	CTCAGCTCGCAGACAGAAACA	
Stat3	20848	Forward	CAATACCATTGACCTGCCGAT	109
		Reverse	GAGCGACTCAAACTGCCCT	
Stat4	20849	Forward	TGGCAACAATTCTGCTTCAAAAC	225
		Reverse	GAGGTCCCTGGATAGGCATGT	
Nfkb1	18033	Forward	ATGGCAGACGATGATCCCTAC	111
		Reverse	TGTTGACAGTGGTATTTCTGGTG	
Nfkb2	19697	Forward	AGCGCGGGGACTATGACTT	121
		Reverse	GCCCGGTTATCAAAAATCGGAT	
Ctnnb1	12387	Forward	ATGGAGCCGGACAGAAAAGC	108
		Reverse	CTTGCCACTCAGGGAAGGA	

### Assessment of Periphery Immunomodulations

The obtained blood samples were separated to plasma and lymphocytes. Lymphocytes were stained (surface and intracellular) to determine Treg^+^ cells CD4^+^CD25^+^FOXP3^+^ and Th1 CD3^+^CD4^+^CD25^−^ population in treated groups in comparison to PBS. First, cells were stained by Percp-Anti-CD3, FITC-Anti-CD4, and PE-Anti-CD25 (Biolegend, USA) for 30 min on ice. Then it was fixed, permeabilized and stained by Alexa Fluor 647- anti- Foxp3 (Biolegend, USA) for 30 min on ice. Finally cells were examined by BD Accuri C6. Later, plasma samples were utilized to determine periphery TGF-β and IL-12 cytokines by ELISA kits according to manufacturer instructions (Lington Biosciences, China).

### Statistical analysis

Statistical analysis was performed using GraphPad Prism 6.0 for at least three independent experiments. Data were analyzed by paired student’s t-test and one-way analysis of variance (ANOVA). The significant difference level was set at p < 0.05.

### Data availability

Authors declare that all data generated or analyzed during this study are included in this published article (and its Supplementary Information files). If any interest needs more details please contact corresponding editor by private email.

## Electronic supplementary material

supplementary information
