# Supplementary material for: 1-Methyl-D-tryptophan Reduces Tumor CD133+ cells, Wnt/β-catenin and NF-κβp65 while Enhances Lymphocytes NF-κβ2, STAT3, and STAT4 Pathways in Murine Pancreatic Adenocarcinoma
Source: Sci Rep. 2018 Jun 29;8:9869. doi: 10.1038/s41598-018-28238-8 (PMC6026162; doi:10.1038/s41598-018-28238-8)
Supplement: Supplementary file 1 — supplementary information [file 41598_2018_28238_MOESM1_ESM.pdf]

Full Title: 1-Methyl-D-tryptophan Reduces Tumor CD133^{+} cells, Wnt/β-catenin and NF-κβp65 while Enhances Lymphocytes NF-κβ2, STAT3, and STAT4 Pathways in Murine Pancreatic Adenocarcinoma

Running Title: 1-MT Prohibited Tumorgensis Signaling Pathways

Murad Alahdal[1,2], Yun Xing[1], Tingting Tang[1], Liang Jin[1†]

Affiliations

1. State Key Laboratory of Natural Medicines, Jiangsu Key Laboratory of Drug Ability of Biopharmaceuticals，Jiangsu Key Laboratory of Drug Screening, School of life Science and Technology, China Pharmaceutical University.
2. Medical Laboratory department, Faculty of Medicine and Health Sciences, Hodeidah University, Yemen.

† Liang Jin corresponding author: State Key Laboratory of Natural Medicines, Jiangsu Key Laboratory of Drug Ability of Biopharmaceuticals，Jiangsu Key Laboratory of Drug Screening, School of life Science and Technology, China Pharmaceutical University.

Address of Corresponding Author Liang Jin: Tongjia Xiang 24, Nanjing, 210009, PR China. Tel: +86 25 83271242. Fax: +86 25 83271242. E-mail: liangjin1975@cpu.edu.cn

Supplementary information

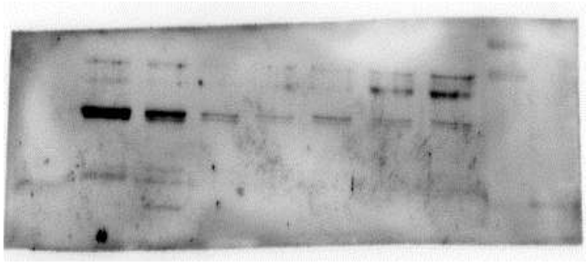

Image (1): TFG-β

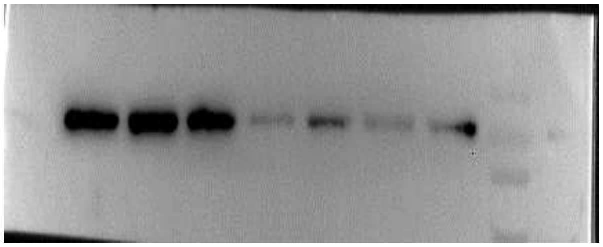

Image (2): PDL-1

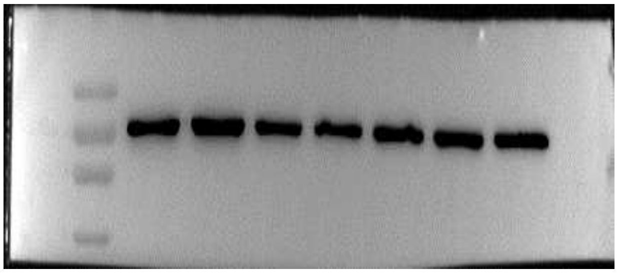

Image (3): GAPDH -1

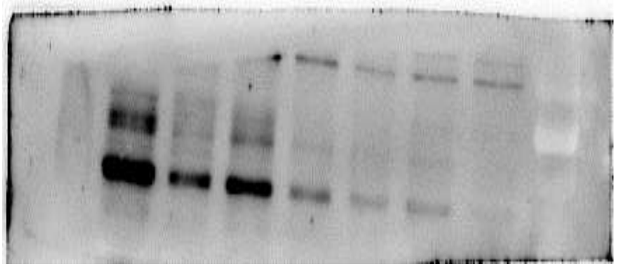

Image (4): NF-κβ2

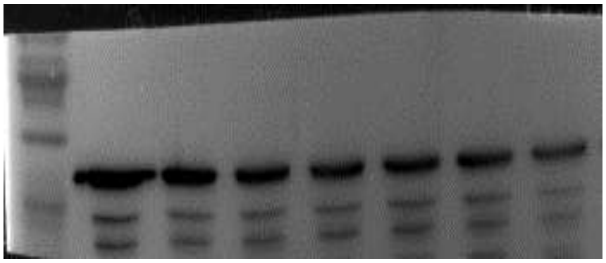

Image (5): GAPDH -2

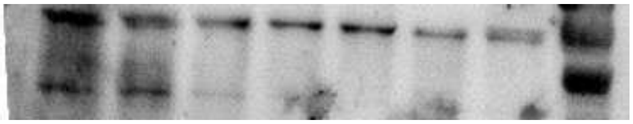

Image (6): IDO

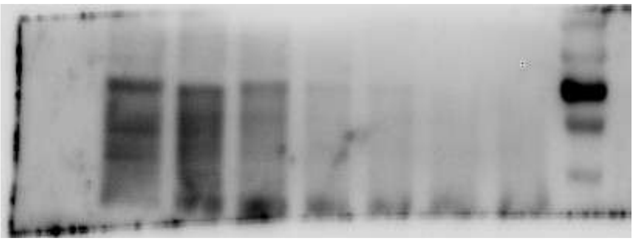

Image (7): β-catenin

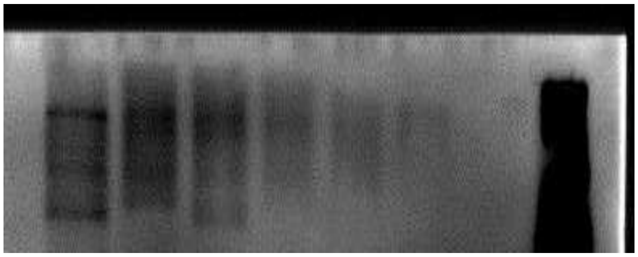

Image (8): CD133

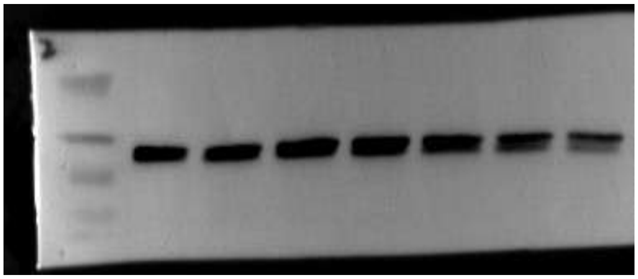

Image (5): GAPDH -3

Supple.Figure1: Here, we listed the original western blot photos, besides some confirmative experiments

1. Lymphocytes proliferation

The proliferation of Th1 and CTL under the effect of 1-MT inhibitor and tumor autologous antigens was performed to explore the role of 1-MT in response to TL vaccination. However, tumor bearing mice were treated three times in the presence of control groups, and then all mice were sacrificed to obtain splenocytes single cell suspension. Later, lymphocytes were separated by mouse Ficoll-Paque and centrifuged; next cells were stained by CFSE 5mM (0.5ul/1x10^7 cells) for 10min at room temperature. Then 1x10^6 cells /well were triply cultured concomitantly with ConA (5ug/ml), PBS, Tumor lysate 20ug/ml, and DC loaded Tumor lysate (1x10^5/ well) for 72 hours. Later, cells were collected, washed and blockade to reduce mismatching staining, and then surface markers of harvested cells were stained by anti-mouse Percp anti-CD3, FITC-anti-CD4, and PE- anti-CD8 for 30 min on ice, and then washed twice by FACS buffer, and evaluated by BD C6 Flowcytometer.

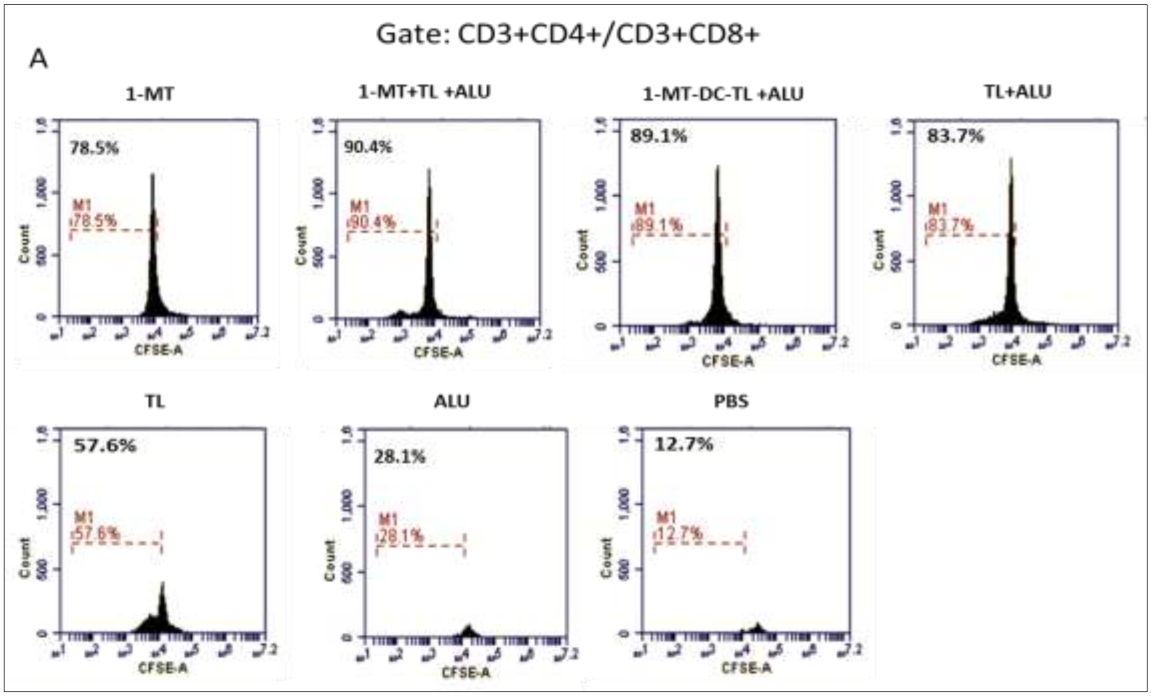

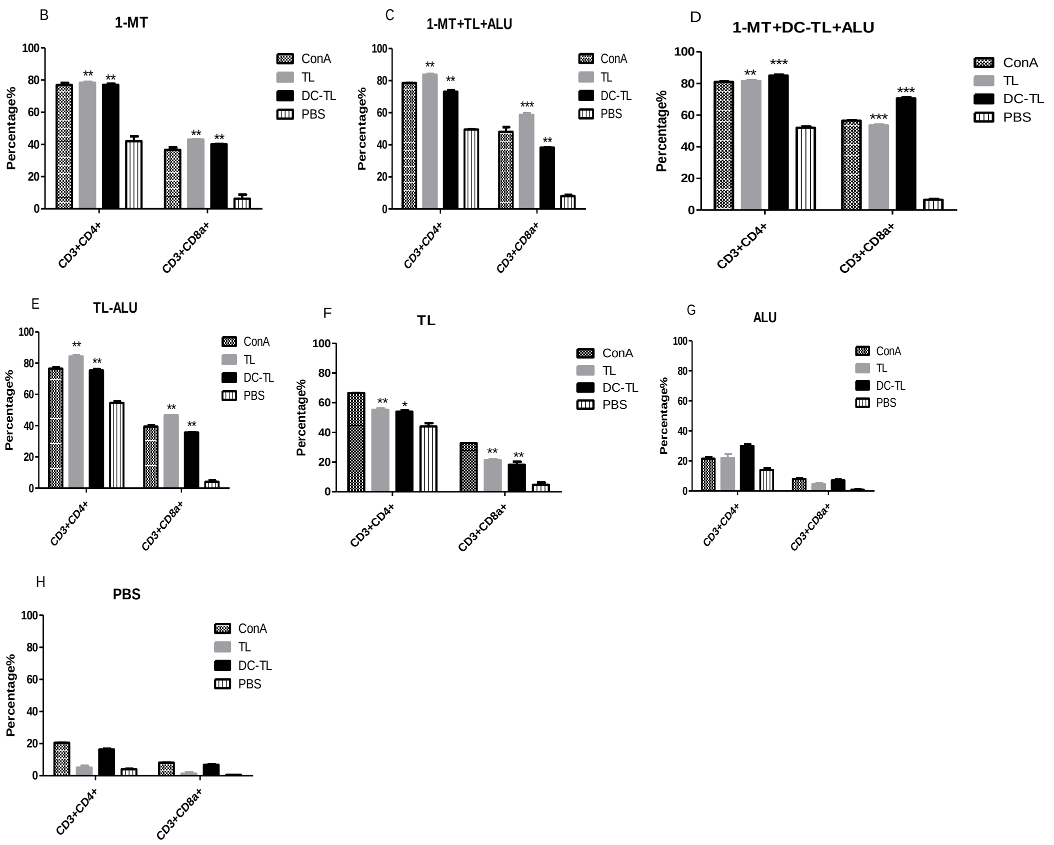

Supple. Figure2: The proliferation of Th1 and CTL under the effect of 1-MT inhibitor and tumor autologous antigens was performed to explore the role of 1-MT in response to TL vaccination. However, tumor bearing mice were treated three times in the presence of control groups, and then all mice were sacrificed to obtain splenocytes single cell suspension. Later, lymphocytes were separated by mouse Ficoll-Paque and centrifuged; next cells were stained by CFSE 5mM (0.5ul/1x107 cells) for 10min at room temperature. Then 1x106 cells /well were triply cultured concomitantly with ConA (5ug/ml), PBS, Tumor lysate 20ug/ml, and DC loaded Tumor lysate (1x105/ well) for 72 hours. Later, cells were collected, washed and blockade to reduce mismatching staining, and then surface markers of harvested cells were stained by anti-mouse Percp anti-CD3, FITC- anti-CD4, and PE- anti-CD8 for 30 min on ice, and then washed twice by FACS buffer, and evaluated by BD C6 Flowcytometer. Results demonstrated that Tumor lysate (TL) and DCs loaded TL in the presence of 1-MT significantly enhanced Th1 and CTL cells proliferation in the 1-MT, 1-MT+TL+ALU, and 1-MT+DCs-TL groups in comparison to PBS group (Fig2.A) According to $CD3^{+}CD8a^{+}$ and $CD3^{+}CD4^{+}$ gate. Meanwhile, separated $CD3^{+}CD4^{+}$ gate ensured that tumor lysate and DCs-TL significantly enhanced Th1, and CTLs proliferation in the 1-MT treated groups (Fig2. B, C, D) in comparison to PBS group, while a response of CTLs

in TL-Alum and Alum groups were noted lower than other tested groups (Fig2. E, G). These results showed a potential of tumor lysate vaccine in the presence of IDO inhibitor to enhance proliferation of inflammatory lymphocytes in the immunized groups and ascribing of provoking inflammatory immunomodulations in the immunized animal models.

2. Tumor cells cytotoxicity

LDH (lactate dehydrogenase) release assay was performed to ensure that tumor antigen was effectively recognized, and CTL cells efficiently promoted tumor cytotoxicity responses. Tumor bearing mice which received vaccine were used to obtain splenocytes which used as an effector cells against pancreatic adenocarcinoma Pan02 cell line as a target cells. Splenocytes were activated by incubation with TAAs (10µg/ml to $1x10^6$ spleen cells), and DCs loaded TL ($1x10^5$ DCs to $1x10^6$ spleen cells) for 24 hours. Later, in the presence of 1-MT, tumor cells and activated splenocytes were mixed and plated into 96 wells plate upon different ratios (T: E) 1:1, 1:5, 1:10, and 1:20 with PBS and Cytoxan (CTX) (Sandoz, USA) as a positive control. Then cells were incubated for 24 hours at 37°C. Later, wells were mixed with 10ul/well of cck8 (Bimake, China), and then formazan was measured by microplate reader at absorbance 450nm. Finally, OD450 was calculated according to the following equation:

Cell viability% = (mixed cells OD- blank OD/ control cells OD - blank OD)*100 (3-1)

Cell apoptosis % = 100% - Cell viability % (3-2)

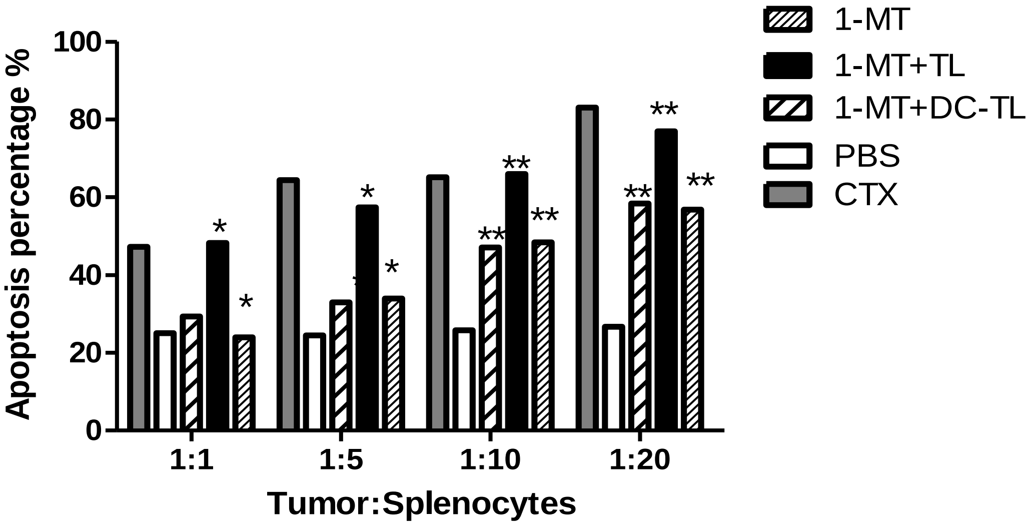

Supple.Figure3: Splenocytes were significantly activated by TL and DC-TL in the presence of 1-MT, which significantly enhanced splenocytes activation. Thereafter, activated spleen cells were introduced to Pan02 cell line in different tested ratios in the presence of positive and negative controls. Results showed that 1-Mehty-D-tryptophan significantly increased tumor cells apoptosis in comparison to PBS group, while the efficacy of TL was noted highly significant than DC-TL in this experiment. ** (*P*<0.001), and * (*P*<0.05)
